# Retraction Note: TRIB2 confers resistance to anti-cancer therapy by activating the serine/threonine protein kinase AKT

**DOI:** 10.1038/s41467-023-40050-1

**Published:** 2023-07-19

**Authors:** Richard Hill, Patricia A. Madureira, Bibiana Ferreira, Inês Baptista, Susana Machado, Laura Colaҫo, Marta dos Santos, Ningshu Liu, Ana Dopazo, Selma Ugurel, Angyal Adrienn, Endre Kiss-Toth, Murat Isbilen, Ali O. Gure, Wolfgang Link

**Affiliations:** 1 Department of Biomedical Sciences and Medicine (DCBM), University of Algarve, Campus de Gambelas, Faro, 8005-139 Portugal; 2 Centre for Biomedical Research (CBMR), University of Algarve, Campus de Gambelas, Faro, 8005-139 Portugal; 3 Brain Tumour Research Centre, Institute of Biomedical and Biomolecular Sciences, University of Portsmouth, PO1 2DT Portsmouth, UK; 4 Bayer AG, Drug Discovery Oncology Research, Berlin, D-13342 Germany; 5 Genomics Unit, Centro Nacional de Investigaciones Cardiovasculares (CNIC), Madrid, 28029 Spain; 6 Department of Dermatology, University Hospital Essen, Essen, 45147 Germany; 7 Department of Cardiovascular Science, University of Sheffield, Sheffield, S10 2RX UK; 8 Department of Molecular Biology and Genetics, Bilkent University, Ankara, 06533 Turkey; 9 Algarve Biomedical Center (ABC), University of Algarve, Campus de Gambelas, Faro, 8005-139 Portugal

Retraction to: *Nature Communications* 10.1038/ncomms14687, published online 09 March 2017

The authors have retracted this article as it has come to their attention that several images were inappropriately processed and duplicated in multiple figures. In particular, the data were duplicated, and in some cases inverted, across several panels in Figures 2c, 2b, 3d and Supplementary Figure 5. Erroneous data were also included in Figure 2e, Supplementary Figure 1 and Supplementary Figure 8. We apologize to the scientific community for any confusion this article may have caused. Richard Hill, Patricia Madureira, Bibiana I. Ferreira, Susana Machado, Ana Dopazo, Selma Ugurel, Endre Kiss-Toth, Murat isbilen and Wolfgang Link agree with this retraction. Inês Baptista, Laura Colaço, Marta dos Santos, Ningshu Liu, Angyal Adrienn and Ali O. Gure have not responded to correspondence from the Publisher about this retraction.

